# The application of plasma circRAD18 in the prediction of gestational diabetes mellitus (GDM) and its adverse effects

**DOI:** 10.1186/s12884-024-06302-8

**Published:** 2024-04-24

**Authors:** Nanying Ni, Lina Li, Mei Xiao, Fengqin Yu

**Affiliations:** Department of Obstetrics and Gynecology, Nanjing Jinling Hospital, No. 34, Bid No. 34, Yanggongjing, Qinhuai District, Nanjing City, Jiangsu Province 210002 China

**Keywords:** circRAD18, Gestational diabetes mellitus, Hypertension

## Abstract

**Background:**

In cancer biology, circRAD18 promotes glucose metabolism, potentially indicating its involvement in glucose metabolism-related disorders, such as gestational diabetes mellitus (GDM). The present study investigated the predictive role of circRAD18 in GDM and its potential adverse effects.

**Methods:**

A total of 482 women who intended to get pregnant in short-term were enrolled. For those who successfully conceived, plasma samples were collected and followed up until delivery to monitor the occurrence of GDM and its associated adverse events. The accumulation of circRAD18 in plasma was analyzed using RT-qPCR. GDM-free curves and ROC curves were plotted to assess the predictive value of plasma circRAD18 for GDM.

**Results:**

After admitting 482 female patients, 388 of them achieved pregnancy within half a year. During the follow-up period, 52 cases were diagnosed with GDM. Compared to non-GDM group (*n* = 336), the GDM group (*n* = 52) had a lower accumulation level of circRAD18 on the day of pregnancy confirmation. In addition, low levels of circRAD18 accumulation on that day distinguished potential GDM patients from non-GDM cases. The 388 cases were divided into high and low circRAD18 level groups (*n* = 194). GDM-free curve analysis showed that patients in the low circRAD18 level group had a higher incidence of GDM compared to the high level group (43/194 vs. 9/194). A close association was found between low levels of plasma circRAD18 and hypertension, but not premature delivery, intrauterine death, malformation, intrauterine infection, miscarriage, macrosomia or intrauterine distress.

**Conclusion:**

The reduction in the accumulation of plasma circRAD18 is predictive of GDM and hypertension in pregnant women.

## Introduction

During pregnancy, hormones produced by placenta, including human placental lactogen, estrogen and cortisol, may block insulin, resulting in insulin resistance [1–3]. This condition is known as gestational diabetes mellitus (GDM). Worldwide, approximately 2–10% of pregnant women are affected by GDM, and the incidence is increasing. This is mainly due to the rise in obesity, modern lifestyles, and older maternal age [4, 5], leading to a high prevalence of GDM in high-risk populations, exceeding 30% [6, 7]. To control blood sugar, patients with GDM typically adhere to insulin injections, scheduled physical activity, and special meal plans [8]. However, numerous complications may persist in these patients, particularly those with obesity or of advanced maternal age [9]. Consequently, alternative approaches are required to enhance pregnancy outcomes among individuals with GDM.

Currently, early detection or prediction of GDM remains a critical aspect of clinical practices to ensure a safe delivery [10–12]. Despite the development of several biomarkers, such as high-sensitive C-reactive protein (hs-CRP), albumin, creatinine and uric acid, their predictive capabilities are limited due to low sensitivity/specificity [13]. Furthermore, these biomarkers often vary among different populations [13, 14]. Although most circular RNAs (circRNAs) lack coding capacity, the remainder can encode small peptides. These circRNAs interact with miRNAs and proteins to regulate human diseases, such as trophoblast cell dysfunction and inflammation [15], which are associated with GDM [16, 17]. Therefore, circRNAs hold significant potential as biomarkers for GDM. It has been established that circRAD18 promotes glucose metabolism in cancer biology [18], suggesting its involvement in glucose metabolism-related disorders, including GDM. Therefore, the present study aimed to assess the value of circRAD18 in predicting GDM and its associated adverse events.

## Materials and methods

### Enrollment of participants

Between May 2019 to May 2020, a total of 482 women with a plan of pregnancy in short-term were enrolled at Nanjing Jinling Hospital, a Grade three comprehensive public hospital. After obtaining approval from the hospital’s Ethics Committee and informed consent, the participant, who were ≥ 18 year old, had a time from their last menstrual period ≥ 1 month, and had confirmed pregnancy by β-hcg testing, were included. Additionally, these participants and their family members had no history of diabetes. Participants with severe diseases, infections, metabolic diseases, mental problems, and any other clinical disorders that were not suitable for pregnancy were excluded. Infertility due to male factors was also excluded by checking the physiological status of their male partners. The participants were asked to take home pregnancy tests every week and 388 cases achieved pregnancy within half a year after admission. In all cases, pregnancy was detected 3 weeks after conception. The Ethics Committee approval number is NO.56,332.

### Follow-up

The 388 cases of pregnant women were followed up until delivery to monitor the occurrence of GDM and its complications. Before 24 weeks, GDM was diagnosed using the WHO criteria, which defines GDM as fasting blood glucose value ≥5.1 mmol/L and ＜7.0mmol/L. At 24–28 weeks, GDM was diagnosed through the Oral Glucose Tolerance Test (OGTT), in which at least one of three criteria must be met: (1) fasting ≥ 95 mg/dL(5.1 mmol/L); (2) 1 h ≥ 180 mg/dL(10 mmol/L); (3) 2 h ≥ 155 mg/dL(8.5 mmol/L). Other complications, including hypertension, premature delivery, intrauterine death, malformation, intrauterine infection, miscarriage, macrosomia and intrauterine distress, were diagnosed using standard methods.

### Plasma and RNA preparation

After the pregnancy confirmation at the aforementioned hospital, all pregnant women were asked not to consume any food and drink overnight (from 8 pm to 8 am). ON the following day, a vein on each participant’s arm was located, and the surrounding skin was thoroughly cleaned using alcohol swabs. Then, a thin and hollow needle was used to withdraw blood into EDTA tubes. After that, all tubes underwent centrifugation at 1,200 g for 15 min, followed by the collection of supernatant (plasma). Plasma samples were mixed with TRIzol reagent (Invitrogen) at a ratio of 1:10 (weight/volume) to extract total RNAs. To achieve complete cell lysis, the tissue and TRIzol mixture were incubated at room temperature for 20 min. After that, centrifugation at 12,000 g was carried out for 15 min to collect the supernatant and remove cell debris. Next, chloroform was mixed with the supernatant at a ratio of 1:4 (volume/volume). After vortexing, centrifugation at 12,000 g was carried out for 10 min to isolate the supernatant containing RNA. Finally, 50% methanol was used to precipitate RNA, and the gDNA was removed using DNase (RNase-free, Promega).

### RT-qPCR

To prepare cDNA samples, the MLV reverse transcriptase (Promega) was used with RNA as the template. if RNA samples exhibited low purity and/or integrity, RNA preparation was repeated. After that, the expression of circRAD18 was detected through qPCRs with 18 S rRNA as the internal control. Ct values were collected and normalized using the 2-delta delta method.

### Statistical analysis

Two groups were compared using an unpaired t test to determine statistical significance. Using the median plasma level of circRAD18 on the day of pregnancy confirmation as the cutoff value, the 388 pregnant women were divided into two groups based on plasma circRAD18 levels (high and low, *n* = 194). GDM-free curves were plotted for both groups, utilizing follow-up data and comparing them using the log-rank test. The predictive value of the plasma level of circRAD18 on the day of pregnancy confirmation for GDM was evaluated using an ROC curve, in which true positive and negative cases were defined as potential GDM and non-GDM cases, respectively. A *P*-value less than 0.05 was considered statistically significant.

## Results

### Follow-up results

Among the 482 females, 388 of them achieved pregnancy within half a year after admission. There patients were closely monitored until delivery, and a total of 52 cases of GDM were diagnosed. Additionally, 18 cases of hypertension, 29 cases of premature delivery, 14 cases of intrauterine death, 10 cases of malformation, 6 cases of intrauterine infection, 22 cases of miscarriage, 31 cases of macrosomia, and 13 cases of intrauterine distress were also diagnosed.

### Comparison of the accumulation of plasma circRAD18 between GDM and non-GDM groups

**Fig. 1:**
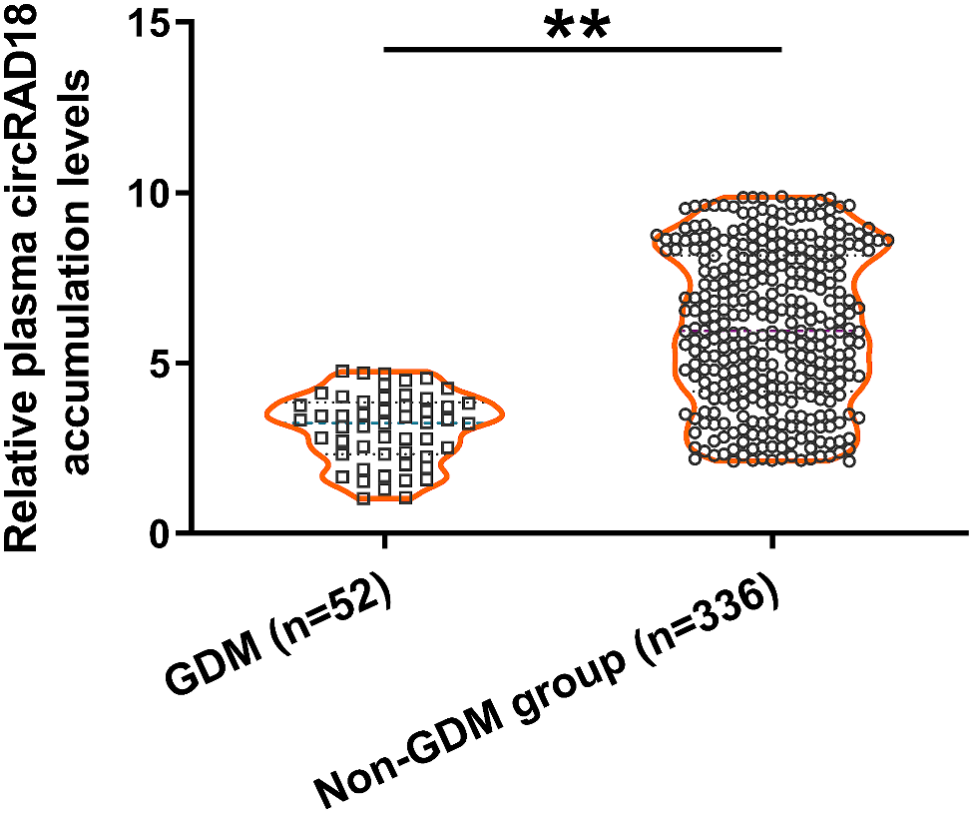
Comparison of the accumulation of plasma circRAD18 between GDM and non-GDM groups. Plasma samples obtained from all 388 cases of pregnant women were used to isolate circulating RNA samples, which were subjected to RT-qPCR to determine the accumulation of circRAD18. **p < 0.01

Plasma samples obtained from all 388 cases of pregnant women were used to isolate circulating RNA samples, which were then subjected to RT-qPCR to determine the accumulation of circRAD18. During the follow-up period, 52 cases of GDM were diagnosed. When compared to the non-GDM group (*n* = 336), a significantly lower accumulation level of circRAD18 was observed on the day of admission in the GDM group (Fig. 1, *p* < 0.01).

### The prediction of GDM using plasma circRAD18 as a biomarker

**Fig. 2:**
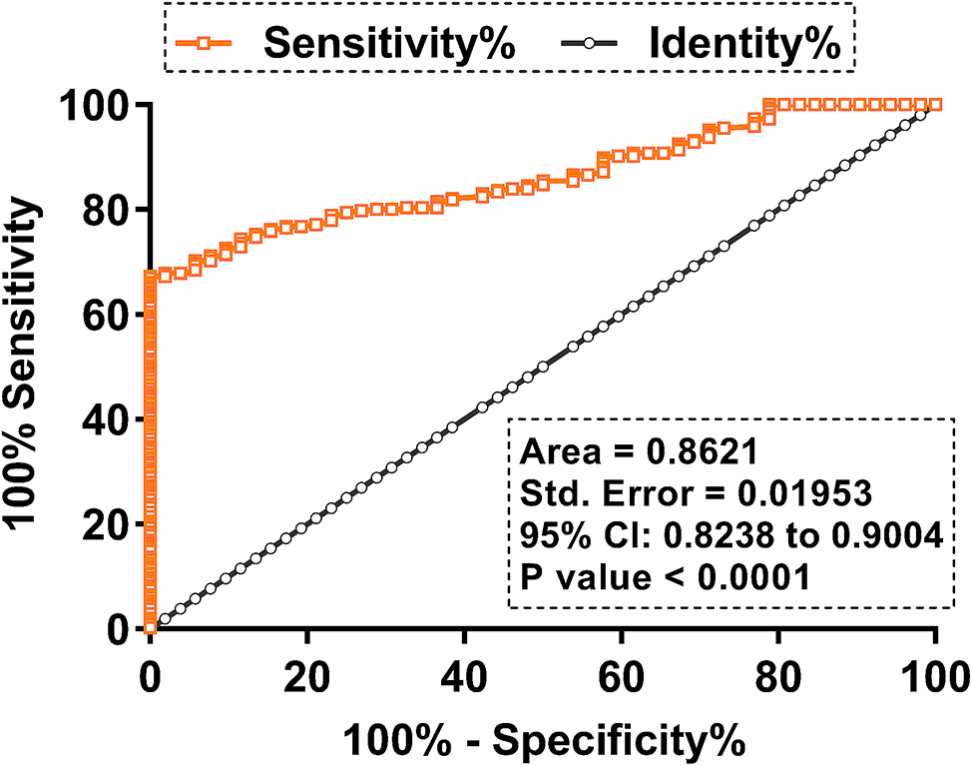
The prediction of GDM using plasma circRAD18 as a biomarker. The prediction value of plasma level of circRAD18 on the day of the confirmation of pregnancy for GDM was explored with ROC curve, in which true positive and negative cases were the potential GDM and non-GDM cases, respectively

The predictive value of plasma level of circRAD18 on the day of pregnancy confirmation for GDM was explored using ROC curve, in which true positive and negative cases were the potential GDM and non-GDM cases, respectively. On the day of pregnancy confirmation, distinguished potential GDM patients demonstrated significantly lower levels of circRAD18 accumulation compared to non-GDM cases (Fig. 2).

### The association between plasma circRAD18 and GDM

**Fig. 3:**
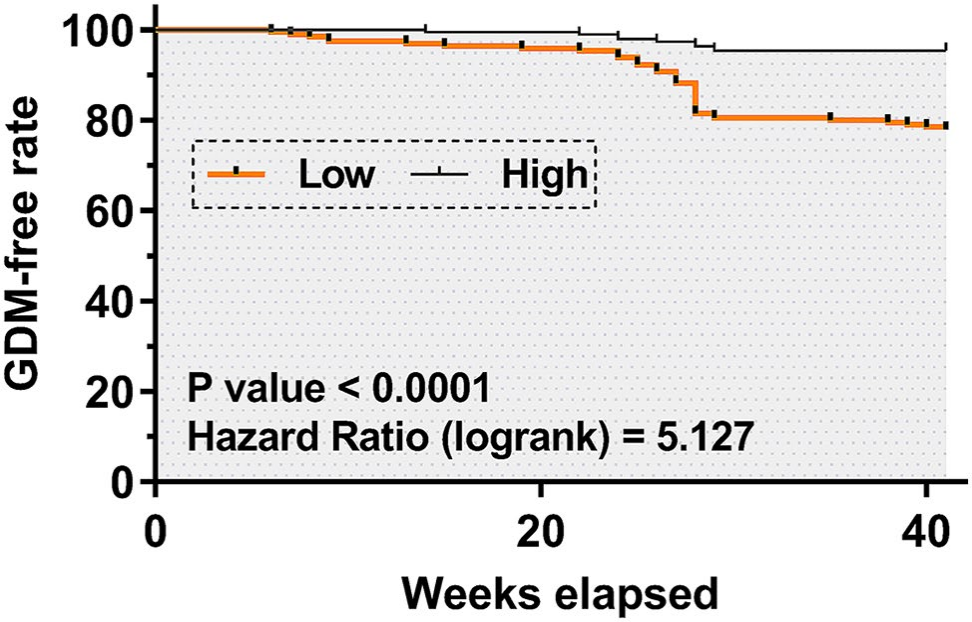
The association between plasma circRAD18 and GDM-related complications. Using the median plasma level of circRAD18 on the day of the confirmation of pregnancy as the cutoff value, the 388 pregnant women were divided into two plasma circRAD18 levels groups (high and low, *n* = 194). GDM-free curves were plotted for both groups. Log-rank test was applied for the comparison

Using the median plasma level of circRAD18 on the day of pregnancy confirmation as the cutoff value, the 388 pregnant women were divided into two plasma circRAD18 levels groups (high and low, *n* = 194). The GDM-free curves were plotted for both groups, and the analysis of GDM-free curve showed that the patients in the low circRAD18 level group had a higher incidence of GDM compared to the high level group (43/194 vs. 9/194) (Fig. 3).

### The association between plasma circRAD18 and GDM-related complications

The incidences of GDM-related complications were compared between individuals with high and low levels of plasma circRAD18. A close association was observed between low levels of plasma circRAD18 and hypertension, but not premature delivery, intrauterine death, malformation, intrauterine infection, miscarriage, macrosomia, and intrauterine distress (Table 1).

**Table 1:** The association between plasma circRAD18 and GDM-related complications

Complications	Cases	High (*n* = 194)	Low (*n* = 194)
Hypertension	18	4	14**
Premature delivery	29	13	16
Intrauterine death	14	6	8
Malformation	10	5	5
Intrauterine infection	6	4	2
Miscarriage	22	10	12
Macrosomia	31	14	17
Intrauterine distress	13	5	8

**p < 0.01

## Discussion

In the present study, we analyzed the accumulation of circRAD18 in the plasma of pregnant women and also examined the role of RNA accumulation in predicting GDM and its associated complications. Our findings suggest the potential application of a novel biomarker in clinical practice for predicting GDM.

CircRAD18 expression is elevated in papillary thyroid cancer, interacting with glucose metabolism pathways to enhance glucose uptake and utilization, thereby promoting tumor progression [18]. This suggests that circRAD18 may also be involved in other glucose metabolism-related clinical conditions, such as GDM. Therefore, we aimed to determine plasma circRAD18 levels in pregnant women prior to the development of GDM. Our findings indicate that plasma circRAD18 accumulation is reduced in women at risk of developing GDM compared to those at risk of non-GDM. Giving circRAD18’s role in promoting glucose metabolism, we speculated that reduced circRAD18 accumulation during pregnancy may limit glucose utilization, potentially increasing the risk of developing GDM. However, this hypothesis requires validation through animal model experiments, which can be challenging to conduct.

Many efforts have been made to improve the early prediction of GDM. For instance, adiponectin, sex hormone-binding globulin and follistatin-like-3 can be detected at 11 to 13 weeks to predict GDM [11]. In this study, we observed that low levels of circRAD18 accumulation on the day of the confirmation (before 3 weeks) of pregnancy distinguished potential GDM patients from non-GDM cases, which we believe is a significant finding. Additionally, during the follow-up period, patients in the low circRAD18 level group experienced a higher incidence of GDM compared to the high level group. Therefore, low accumulation levels of circRAD18 in plasma may serve as a potential indicator of GDM. However, this conclusion should be further validated through additional clinical trials. It is important to note that polycystic ovary syndrome (PCOS) is a risk factor for sub-fertility and abnormal glucose tolerance testing results in both non-pregnant and pregnant individuals, as well as GDM [19]. However, a limitation of our study is the lack of data on the presence of PCOS in our study population.

Interestingly, it has been observed that decreased expression of circRAD18 is specifically linked to hypertension, rather than other GDM-related complications. It is well documented that elevated blood glucose levels significantly increase the risk of hypertension [20]. The underlying reasons for this are multi-fold: damage to the vascular endothelium, spasms in the small arteries throughout the body, and ultimately, an increase in blood pressure due to physical factors unique to each individual. Furthermore, we postulate that the decreased accumulation of circRAD18 may indirectly contribute to hypertension through its impact on glucose metabolism.

In conclusion, circRAD18 is downregulated in GDM and its reduced levels in the blood plasma could potentially serve as a biomarker for predicting GDM and its associated complications.

## Data Availability

The sets of data analyzed in the study can be obtained from the author upon request, in a reasonable manner.
